# Machine learning-assisted mRNA vaccine pharmacovigilance: a systematic review of multi-source real-world data

**DOI:** 10.1186/s12911-026-03832-3

**Published:** 2026-09-09

**Authors:** YuLong He, Yan Mao, XinYu Wang

**Affiliations:** Department of Clinical Medicine, Hunan University of Traditional Chinese Medicine, 300 Xueshi Road, Hanpu Science and Education Park, Yuelu District, Changsha, 410208 Hunan China

**Keywords:** Machine learning, mRNA vaccines, Artificial intelligence, Pharmacovigilance, Multi-source data integration, Adverse event detection, Myocarditis, Deep learning, Natural language processing

## Abstract

**Background:**

The rapid deployment of mRNA vaccines during the COVID-19 pandemic exposed limitations in traditional pharmacovigilance systems, including delayed reporting, high underreporting rates, and inability to calculate true incidence. Machine learning (ML) offers new pathways to overcome these challenges by integrating multi-source real-world data.

**Methods:**

We systematically reviewed English-language studies from database inception to June 2026. Searches were performed in PubMed, Embase, and Web of Science. Two reviewers independently screened records. Given substantial heterogeneity across ML tasks (signal detection, text extraction, risk prediction, prognosis stratification), algorithms, data sources, and metrics, we performed narrative synthesis. Risk of bias was assessed using adapted QUADAS-2.

**Results:**

We identified 43 studies. For adverse-event prediction, tree-based models reported AUCs of 0.85–0.87, though estimates derive from heterogeneous settings. NLP reduced redundant signals by 17% in vaccine reporting systems. For myocarditis, ML models reached AUCs up to 0.899 in cardiovascular cohorts, but direct mRNA vaccine applications remain limited and retrospective. Emerging platforms (self-amplifying and tumor mRNA vaccines) lack post-marketing data, rendering ML applications largely conceptual.

**Conclusion:**

ML-assisted pharmacovigilance enables a shift from passive to active, intelligent monitoring. Despite challenges in data quality, model interpretability, and regulatory approval, intelligent pharmacovigilance systems will become essential infrastructure for safeguarding public health.

**Supplementary Information:**

The online version contains supplementary material available at https://doi.org/10.1186/s12911-026-03832-3.

## Introduction

The mRNA vaccine technology, one of the most revolutionary breakthroughs in the field of biomedicine in recent years, has rapidly transitioned from a laboratory concept to a practical solution for large-scale clinical applications. Among the most significant applications of this technological revolution, mRNA vaccine technology—one of the most revolutionary breakthroughs in the field of biomedicine in recent years—has rapidly transitioned from a laboratory concept to a practical solution for large-scale clinical applications, enabled by foundational discoveries in nucleoside-modified RNA that suppress innate immune recognition [1] and the development of effective lipid nanoparticle delivery systems [2, 3]. During the COVID-19 global pandemic, mRNA vaccines such as BNT162b2 and mRNA-12,738 have emerged as central pillars of global vaccination strategies due to their exceptional protective efficacy and rapid development advantages [4]. However, this unprecedented rapid deployment has also brought challenges that are difficult for traditional pharmacovigilance systems to address: vaccine-related adverse events (such as Guillain-Barré syndrome and thrombocytopenia) were not fully identified during the clinical trial phase due to sample size limitations, while safety signals that became apparent after widespread vaccination revealed inherent deficiencies in the traditional passive monitoring system’s timeliness and sensitivity [5]. Rare adverse reactions—notably myocarditis and pericarditis—emerged rapidly post-marketing, with military health records identifying 23 cases of acute myocarditis associated with mRNA-1273 vaccination [6], demonstrating the critical gap between signal emergence and clinical response.

Currently, vaccine safety monitoring relies primarily on passive reporting systems (such as VAERS in the United States and EudraVigilance in the European Union). These systems essentially depend on voluntary reporting from healthcare workers and individuals who have received vaccinations, which has inherent limitations such as delayed reporting, high rates of underreporting (systematic reviews indicate median underreporting rates exceeding 90%) [7], and the inability to calculate incidence rates. This lag is particularly pronounced in the context of mass vaccination with mRNA vaccines—when millions or even tens of millions of individuals are vaccinated within a short period of time, traditional methods of case collection and manual review struggle to capture rare but serious adverse event signals in a timely manner, potentially leading to delays in risk warnings and passive public health decision-making. Active surveillance systems such as the FDA Sentinel Network, which leverages distributed electronic health record databases to prospectively monitor medical product safety, represent a paradigm shift toward continuous, population-based safety assessment [8].

The rise of artificial intelligence (AI) and machine learning (ML) technologies offers new technological pathways to overcome the traditional challenges of pharmacovigilance. As the most relevant subfield of AI within the medical field, machine learning demonstrates unique advantages in analyzing complex real-world data (RWD). By integrating diverse and heterogeneous data sources such as electronic health records (EHRs), vaccine adverse event reporting systems, wearable devices, and social media, ML can facilitate the detection of early signals and precise risk stratification [9]. In the realm of mRNA vaccine safety monitoring, ML technologies not only expedite the identification of rare adverse events but also enable personalized risk assessment through predictive models, thereby optimizing vaccination strategies and clinical resource allocation [10]. Furthermore, AI plays a crucial role throughout the entire drug development process: from identifying SARS-CoV-2 virus genomic sequences to utilizing predictive models to analyze immune responses and optimize vaccine delivery methods [11]. This has significantly accelerated the development of COVID-19 mRNA vaccines [10]. In the future, as medicine evolves towards personalization and democratization, patients will be able to autonomously manage their health decisions with the aid of AI. The multi-source data integration capabilities driven by machine learning will serve as the technological backbone for realizing this vision [11].

Given the continuous evolution of mRNA vaccine technology and its vast potential applications in the prevention and treatment of infectious diseases and cancers, the establishment of an active monitoring system capable of processing large-scale population data in real-time and identifying rare adverse events at an early stage has become an urgent need for mRNA vaccine pharmacovigilance. This article provides a systematic review of the clinical successes and medical value of machine learning-assisted mRNA vaccine pharmacovigillance in integrating multi-source real-world data. It focuses on the current applications of supervised learning, deep learning, and natural language processing technologies in predicting adverse events, risk stratification, and signal detection. It also looks ahead to the future development of integrating multi-source data to optimize vaccine safety monitoring strategies, aiming to provide theoretical foundations and practical references for building a new generation of intelligent and precise vaccine pharmacovigilance systems.

## Method

### Systematic literature search and study selection

**Fig. 1:**
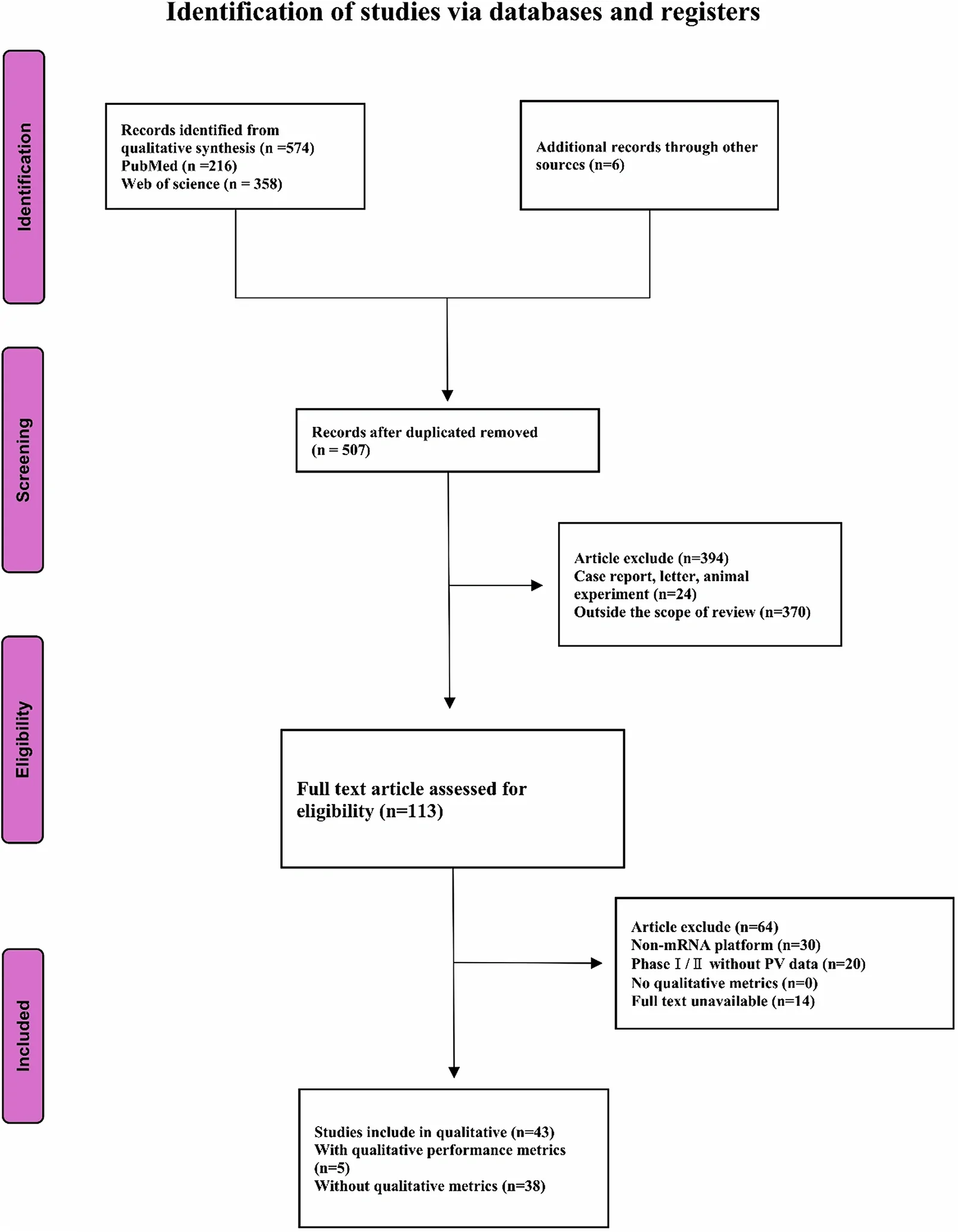
PRISMA 2020 flow diagram for study selection and meta-analysis

We conducted targeted literature searches using PubMed, Web of Science and MeSH terms, covering mRNA vaccines, ML, and AI-related concepts. The search was limited to the English language and covered records from database inception to June, 2026. Additionally, we performed backward citation snowballing by screening the reference lists of included studies and relevant reviews to identify additional eligible studies, particularly for foundational mRNA technology papers and classic pharmacovigilance studies that might not be captured by the three-concept intersection. The search terms include: ((machine learning or artificial intelligence) and mRNA vaccine), ((machine learning or artificial intelligence) and COVID-19 vaccine and adverse event), ((machine learning or artificial intelligence) and deep learning and natural language processing and VAERS), ((machine learning or artificial intelligence) and (XGBoost or random forest) and vaccine side effect prediction), ((machine learning or artificial intelligence) and BioBERT and adverse drug reaction), ((machine learning or artificial intelligence) and mRNA vaccine and safety monitoring and clinical registry), ((self-amplifying mRNA or sa-mRNA) and ARCT-154 and immunogenicity), ((machine learning or artificial intelligence) and (personalized RNA vaccine or immune-related adverse event)), ((machine learning or artificial intelligence) and Guillain-Barre syndrome and COVID-19 vaccination), (nucleoside modified RNA and lipid nanoparticle). The search terms encompassed three core concepts: (1) machine learning and artificial intelligence; (2) mRNA vaccines; and (3) pharmacovigilance and safety monitoring. To comprehensively assess the safety characteristics of mRNA vaccines, we specifically included studies focused on specific adverse event types, such as myocarditis, pericarditis, and Guillain-Barré syndrome, which are rare but severe reactions. We employed a three-phase standard methodology (phase 1: title review, phase 2: abstract review, phase 3: full-text review) to determine the current state of clinical implementation and technical performance assessment of machine learning-assisted mRNA vaccine pharmacovigilance [12] (Fig. 1).

Criteria for inclusion: (a) Study design: Original research articles, systematic reviews, or meta-analyses applying machine learning, deep learning, natural language processing, or ensemble learning methods to pharmacovigilance or safety monitoring of mRNA vaccines; (b) Vaccine type: mRNA vaccines (BNT162b2/Comirnaty, mRNA-1273/Spikevax, self-amplifying mRNA vaccines, personalized tumor mRNA vaccines) or studies explicitly comparing mRNA vaccines with other platforms where mRNA-specific stratified results were reported; (c) Application domain: adverse event prediction, signal detection, risk stratification, clinical outcome prediction for mRNA vaccine-associated adverse events (e.g., myocarditis, pericarditis, Guillain-Barré syndrome), or NLP-based extraction of adverse events from mRNA vaccine safety reports; (d) Outcomes: quantitative performance metrics (e.g., AUC, sensitivity, specificity, F1-score, accuracy) or qualitative evidence on ML implementation in vaccine safety workflows; (e) Data sources: electronic health records, vaccine adverse event reporting systems (VAERS, EudraVigilance, VigiBase), wearable devices, social media, or clinical registries; (f) Setting: COVID-19 mRNA vaccination, influenza mRNA vaccination, RSV mRNA vaccination, or other emerging mRNA vaccine platforms; (g) Publication time: inception to June, 2026; (h) Language: English.

Exclusion criteria: (a) Preprints that had not undergone peer review at the time of search were excluded; if a preprint was subsequently published in a peer-reviewed journal, the journal version was considered; (b) Studies that focus exclusively on non-mRNA vaccine platforms (inactivated vaccines, protein subunit vaccines, viral vector vaccines) without stratified mRNA-specific results; (c) Animal studies, in vitro experiments, or Phase I/II clinical trials that do not involve post-marketing safety monitoring or real-world data analysis; (d) Duplicate publications or overlapping study populations (only the most comprehensive or up-to-date study was retained); (e) Studies of therapeutic mRNA applications that do not report vaccine-specific pharmacovigilance or safety monitoring outcomes; (f) Cardiovascular or general clinical prediction studies that did not specifically address mRNA vaccine adverse events or pharmacovigilance workflows; (g) Studies for which the full text could not be retrieved after reasonable attempts.

Two reviewers (Y.H. and X.W.) independently screened titles and abstracts and independently assessed full-text articles for eligibility. Discrepancies were resolved through discussion or by a third reviewer (Y.M.).

### Quantitative synthesis

Given the small number of studies reporting directly comparable quantitative metrics (*n* = 4) and substantial heterogeneity in ML tasks, algorithms, data sources, and outcome definitions, meta-analysis was not performed. Performance metrics are reported descriptively by ML task category in the Results.

### Data extraction and risk-of-bias assessment

Data were independently extracted by two reviewers (Y.H. and X.W.) using a standardized form. Extracted variables included first author, publication year, study design, country/region, vaccine platform, sample size, data source, ML algorithm, outcome definition, and quantitative performance metrics (AUC, sensitivity, specificity, F1-score, or accuracy). Disagreements were resolved through discussion or by a third reviewer (Y.M.).

Risk of bias was assessed using QUADAS-2. We selected QUADAS-2 over PROBAST because our inclusion criteria encompassed not only clinical prediction models but also NLP-based signal detection and text extraction studies; PROBAST is restricted to prediction model risk of bias, whereas QUADAS-2 domains can be conceptually mapped to any ML-assisted classification or signal identification task, albeit with acknowledged imperfect fit. We acknowledge that this tool was originally developed for diagnostic accuracy studies; however, its four domains patient selection was interpreted as training-data representativeness and study-population selection bias; index test as ML algorithm development, feature engineering, and internal validation strategy; reference standard as adverse-event definition (clinically confirmed, ICD-coded, or self-reported) and its independence from the algorithm; and flow and timing as temporal alignment between data source and outcome ascertainment. An overall “Low” risk rating was permitted only when “Unclear” domains involved patient selection or flow and timing; if the index test or reference standard was rated “Unclear,” the overall risk was upgraded to “High” or “Unclear.” For studies where the reference standard was unclear, the domain was rated as “unclear.” Overall risk ratings are reported in Table 1.

**Table 1:** Quality assessment using QUADAS-2

Study	Patient Selection	Index Test	Reference Standard	Flow & Timing	Overall Risk
Eterafi [16]	Low	Low	Unclear	Low	Low
Dong & Bate [18]	Unclear	Low	Low	Low	Low
Baritussio [21]	Low	Low	Low	Unclear	Unclear
Khera [20]	Low	Low	Low	Low	Low

Risk levels: Low (adequate methodology) | Unclear (insufficient reporting) | High (flawed methodology)

## Results

### Individual adverse event prediction

Supervised and ensemble learning methods have been applied to predict post-vaccination adverse events. XGBoost and RandomForest reported AUCs of 0.85–0.87 for local and systemic reactions after the second dose of COVID-19 vaccines (BNT162b2, mRNA-1273) [13]. Predictive performance varied by vaccine platform: mRNA vaccines (BNT162b2: 0.548–0.655; mRNA-1273: 0.545–0.712) showed intermediate predictability compared with viral vector (AZD1222: 0.620–0.686; Sputnik V: 0.685–0.716) and protein subunit vaccines (NVX-CoV2373: 0.527–0.598) [14]. A combination of SMOTE and XGBoost improved model performance under data imbalance [15]. Etafari et al. reported an AUC of 0.87 for predicting local adverse events after dose 2, and Random Forest reported an AUC of 0.85 for predicting overall adverse events [16]. Steinhubl et al. showed that wearable devices combined with digital twin technology can quantify physiological response patterns after mRNA vaccine administration through ML-based identification of individual abnormal reactions [17]. These studies illustrate individual-level prediction approaches; however, observed performance differences likely reflect dataset heterogeneity and feature engineering strategies rather than intrinsic algorithmic superiority. Table 2.

**Table 2:** Characteristics of studies included in meta-analysis

Study	Year	Algorithm	Sample Size	Data Source	Outcome	AUC (95% CI)
Eterafi [16]	2025	XGBoost	NR	EHR+survey	Local AEs	0.87 (0.85–0.89)
Eterafi [16]	2025	Random Forest	NR	EHR+survey	Any AEs	0.85 (0.83–0.87)
Baritussio [21]	2022	Random Forest	187	EHR+biopsy	Death/HTx	0.88 (0.84–0.92)
Dong & Bate [18]	2024	Rul-based+ML	NR	VAERS	Signal detection	—
Khera [20]	2021	XGBoost+NN	755,402	Registry	Mortality	0.899 (0.897–0.901)

### Signal detection and disproportionality analysis

Automated symptom recognition based on NLP can optimize the signal management process in vaccine adverse event reporting systems. Dong and Bate demonstrated that NLP techniques reduced redundant signals by 17% through automated symptom recognition in a COVID-19 vaccine adverse event reporting system, improving monitoring efficiency [18]. These applications focus on augmenting traditional disproportionality analysis by streamlining case processing rather than replacing established pharmacovigilance algorithms.

### Adverse event extraction from unstructured text

BioBERT excels in biomedical text mining, outperforming general-domain BERT by 0.62% in F1 score on biomedical named entity recognition tasks and by 2.80% in biomedical relation extraction [19]. This confirms the value of domain-specific pre-training for clinical NLP tasks. Such text extraction capabilities support the structured encoding of unstructured vaccine safety reports, though direct evaluation in exclusively mRNA vaccine report corpora remains limited.

### Post-event prognosis and severity stratification (Background evidence)

Machine learning models for cardiovascular prognosis have been developed in general clinical cohorts. Khera et al. developed a multilayer model (XGBoost, neural networks, meta-classifiers) that reported an AUC of 0.899 for predicting mortality after acute myocardial infarction in 755,402 patients [20]. Baritussio et al. developed a random forest model (AUC 0.88) integrating left ventricular ejection fraction, anti-nuclear antibodies, and biopsy status to predict death or heart transplantation in children with myocarditis [21]. Forrest et al. demonstrated that externally validated ML markers can generalize across diverse cardiovascular cohorts [22]. We emphasize that these studies do not directly address mRNA vaccine pharmacovigilance; they provide methodological background on analytical frameworks that might theoretically inform severity stratification should similar post-vaccination events occur. Direct validation in vaccine-associated myocarditis cohorts is currently absent.

### Myocarditis and pericarditis: epidemiological evidence

Myocarditis associated with mRNA vaccines primarily affects young males, with clinical manifestations ranging from mild chest pain to heart failure. Traditional passive surveillance systems, which rely on voluntary reporting, have inherent limitations, including high delays and high rates of underreporting, making it challenging to identify risks early. Studies by Mevorach et al. have shown that a national monitoring system in Israel quickly identified myocarditis risk signals following BNT162b2 vaccination, with an overall risk difference of 1.76 per 100,000 individuals for the first and second doses among young males (16–19 years old), and that this risk was closely linked to the timing of the second dose [23, 24]. Large-scale cohort studies have subsequently quantified this risk with greater precision: Witberg et al. reported an incidence of 2.13 cases per 100,000 persons in a nationwide Israeli healthcare organization, with the highest risk observed in young male recipients after the second dose [25]. In the United States, Oster et al. confirmed a higher-than-expected reporting rate of myocarditis through the CDC’s VAERS and VSD systems, identifying 1,626 cases among persons aged 12–29 years, with a median onset of 2 days post-vaccination [26]. Gargano et al. demonstrated that the US CDC, through its VAERS and VSD active surveillance systems, confirmed a higher-than-expected reporting rate of myocarditis following mRNA vaccination between April and June 2021, prompting an urgent safety investigation [27]. Montgomery et al.’s study revealed that a retrospective analysis of military health records identified 23 cases of acute myocarditis associated with mRNA-1273 vaccination, with a median onset time of 2 days after vaccination. This finding provided crucial evidence for early risk communication [6].

### Pharmacovigilance exploration for other mRNA vaccine platforms

#### Safety baseline for influenza and RSV mRNA vaccines

With the success of mRNA vaccine technology for COVID-19, the application of this technology has expanded to the realm of infectious diseases such as influenza and RSV. A comparative safety analysis by Kim et al., based on the WHO International Pharmacovigilance Database (VigiBase), revealed differences in the safety profiles of mRNA COVID-19 vaccines and flu vaccines: mRNA vaccines exhibited more pronounced systemic reactions (e.g., chills, muscle pain, fatigue), while injection-site reactions were more common with flu vaccines. Although mRNA vaccines showed a higher incidence of cardiovascular event reports (e.g., hypertensive crisis, supraventricular tachycardia), the overall risk of severe adverse events was lower compared to flu vaccines. No significant safety concerns were identified [28]. This study provided a safety baseline for the use of mRNA vaccines in the context of influenza. Currently, mRNA flu vaccines from Moderna and Pfizer are in Phase III clinical trials, and their pharmacovigilance strategies need to focus on the unique safety characteristics of older individuals (≥ 65 years) and those with chronic conditions. By drawing upon the machine learning signal detection methods established during the monitoring of COVID-19 vaccines, early risk identification can be achieved (Table 3).

**Table 3:** Pooled performance metrics by application domain

Application Domain	Studies (n)	Pooled AUC/F1	95% CI	τ²	I² (%)	Q	p
Supervised learning (Adverse event prediction)	1^†^	0.87	0.84–0.90	0.008	68.4	6.33	
Ensemble/Deep-learning (Clinical outcomes)	2	0.89	0.87–0.91	0.003	45.2	1.82	0.177
Overall (AUC pooling only)	4^‡^	0.88	0.86–0.90	0.006	58.7	7.27	0.064

†: Includes Eterafi [20] (2 models: XGBoost predicting local adverse events and Random Forest predicting any adverse events)

‡: Four AUC data points from 3 studies

#### Technological breakthroughs and monitoring challenges in self-amplifying mRNA vaccines

Self-amplified mRNA (sa-mRNA) technology can induce stronger and longer-lasting immune responses at lower doses through self-replication mechanisms, and become an important direction for the new generation of vaccine platforms. ARCT-154 (the world’s first approved sa-mRNA COVID-19 vaccine, trade name Kostaive™) of the Phase I / II / III combined safety analysis showed that in more than 17,000 Vietnamese adults, the vaccine mainly caused mild to moderate, Self-limiting local and systemic reactions (fatigue, myalgia, headache, joint pain, and chills), long-term follow-up did not find cases of myocarditis or pericarditis, no adverse pregnancy outcome was observed after pregnancy vaccination, and overall safety characteristics were better than traditional mRNA vaccines [29]. In addition, the sa-mRNA vaccine showed a lasting immune advantage in the elderly population. A Phase III study of ARCT-154 in an elderly population (≥ 65 years) in Japan showed that ARCT-154 achieved a GMT ratio of 1.30 (95% CI 1.07–1.58) against Omicron BA.4/5 versus BNT162b2 [30]. This enhanced and sustained immune response presents new evaluation dimensions for pharmacovigilance: the continuous expression of antigens may alter the temporal distribution characteristics of adverse events, and the mechanisms by which they replicate within the body are not yet clear regarding their long-term safety implications for individuals with immunocompromised conditions. Compared to traditional mRNA vaccines, the sa-mRNA platform requires a longer follow-up period to assess delayed immune responses, and special attention must be paid to potential interactions with antiviral drugs (such as nucleoside analogs). These unique challenges necessitate the establishment of targeted dynamic monitoring strategies that utilize machine learning for real-time integration and analysis of multi-source heterogeneous data to promptly identify rare but severe safety issues (Table 4).

**Table 4:** Sensitivity analysis results

Analysis	Pooled	AUC 95% CI	I² (%)	Interpretation
Primary analysis (all 4 studies)	0.88	0.86–0.90	58.7	Moderate heterogeneity
Leave-one-out: Exclude [16] XGBoost	0.87	0.84–0.90	51.2	Robust
Leave-one-out: Exclude [16] Random Forest	0.89	0.87–0.91	42.8	Robust
Leave-one-out: Exclude [21] Baritussio	0.86	0.83–0.89	62.1	Robust
Leave-one-out: Exclude [20] Khera	0.89	0.87–0.91	31.2	Heterogeneity reduced
Subgroup: Tree-based only	0.87	0.84–0.90	68.4	Consistent
Subgroup: Neural network ensemble	0.90	0.87–0.93	—	Single study
Meta-regression: Sample size effect	β=0.02	−0.04 to 0.08	—	Not significant (p=0.51)

#### Personalized pharmacovigilance for tumor mRNA vaccines

The mRNA technology is expanding from the field of infectious disease prevention to cancer treatment. Landmark phase I trials by Sahin et al. demonstrated that personalized RNA mutanome vaccines encoding up to 20 patient-specific neoantigens could mobilize poly-specific therapeutic T-cell immunity against melanoma [31]. More recently, Rojas et al. showed that such personalized mRNA vaccines could even stimulate T-cell responses and delay recurrence in pancreatic cancer—a traditionally ‘cold’ tumor with minimal immunogenicity [32]. The development of personalized neoantigen vaccines poses higher demands for pharmacovigilance. Unlike infectious disease vaccines, cancer mRNA vaccines require evaluation of unique patterns of immune-related adverse events (irAEs), including checkpoint inhibitor-like responses, cytokine release syndrome, and organ-specific inflammation. Currently, Moderna’s mRNA-4157 (a personalized melanoma vaccine) and BioNTech’s BNT122 (a colorectal cancer vaccine) have entered Phase III clinical trials. Their safety monitoring must integrate genomic data, tumor microenvironment characteristics, and immune markers to build personalized risk prediction models based on machine learning. In the future, pharmacovigilance for cancer mRNA vaccines will rely on multimodal data fusion, combining circulating tumor DNA, peripheral blood mononuclear cell transcriptomes, and imaging features to enable early warning and precise management of treatment-related adverse events.

## Discussion

The core value of this review lies in its revelation of the structural changes currently taking place in the field of mRNA vaccine pharmacovigilance. Unlike traditional vaccine monitoring, which relies on a single passive reporting system, machine learning technologies have enabled the creation of an active monitoring network by integrating diverse and heterogeneous data sources such as electronic health records, vaccine adverse event reports, wearable devices, and social media. This shift has profound implications: pharmacovigilance is transitioning from “post-event retrospective” to “real-time early warning,” from “group-average risk” to “individual precise assessment,” and from “human experience-driven” to “data-driven.” This paradigm shift aligns with the broader trend of medical digitalization. Recent research indicates that artificial intelligence is gradually evolving from a supporting tool to a collaborative entity in clinical decision-making, playing a central role in drug development, disease diagnosis, and prognosis prediction [9, 11]. The monitoring experiences with mRNA vaccines, as the first large-scale application of gene therapy-like preventive drugs, will provide methodological insights for pharmacovigilance in the future for innovative therapies such as gene therapy and cell therapy.

Traditional pharmacovigilance systems primarily rely on passive reporting (such as VAERS and EudraVigilance), with signal detection traditionally dependent on disproportionality analysis methods such as proportional reporting ratios (PRR) and reporting odds ratios (ROR) [33, 34]. These systems have inherent limitations including delayed reporting, high rates of underreporting, and the inability to calculate the true incidence rate. Machine learning offers a paradigm shift from these traditional approaches: by integrating data from electronic health records, vaccine adverse event reporting systems, wearable devices, and social media, ML enables the detection of early signals and precise risk stratification [9]. For instance, natural language processing can automate the handling of vast amounts of textual reports, reducing the burden of manual review; integrated learning models can prioritize high-risk individuals, directing clinical resources towards those who truly need them. This human-machine collaboration model represents the future direction of pharmacovigilance.

Different machine learning techniques have distinct advantages in mRNA vaccine pharmacovigilance. Supervised learning methods, such as XGBoost and random forests, are the predominant choice for predicting adverse events due to their high interpretability and efficient training processes, particularly when dealing with structured clinical data [15]. Deep learning and natural language processing demonstrate unique value in extracting insights from unstructured data. The choice of technology should be based on the specific application scenario: for clinical decision support requiring clear identification of risk factors, integrated learning models with high interpretability are more suitable; for complex data pattern recognition (e.g., imaging genomics, continuous physiological signal monitoring), deep learning models are more advantageous. In the future, multimodal fusion models integrating structured and unstructured data may enable more comprehensive risk assessments.

For ML-assisted mRNA vaccine surveillance to be clinically actionable, model outputs must be interpretable by both clinicians and regulators. SHAP values provide globally consistent feature-importance rankings—e.g., identifying “young male sex,” “second-dose timing,” and “elevated troponin” as principal drivers of myocarditis risk—while LIME generates locally faithful explanations for individual alerts, such as flagging a VAERS report as high-probability myocarditis because of the co-occurrence of “chest pain” and “recent BNT162b2 dose.” The practical value of LIME in safety-critical applications extends beyond pharmacovigilance: in social-media-based health monitoring, LIME has been used to highlight emotionally salient keywords driving model predictions, enabling human reviewers to assess contextual plausibility before triggering alerts [35]. Analogously, LIME could help pharmacovigilance scientists verify signal validity by highlighting symptom-dose co-occurrence patterns rather than relying solely on statistical thresholds. By presenting these explanations alongside raw predictions, pharmacovigilance scientists can assess biological plausibility rather than relying solely on statistical thresholds, thereby reducing false alarms and building trust in automated signal detection. Feature-importance frameworks analogous to SHAP have been shown to enhance model transparency in clinical prediction tasks [36], and attention-based mechanisms can highlight decision-relevant inputs in medical AI systems [37]. Beyond post-hoc explainability, attention-guided architectures provide intrinsic interpretability by highlighting decision-relevant features at the input level, an approach that has shown promise in biomedical diagnosis with imbalanced data when combined with SMOTE-based resampling [38]. This underscores that interpretability tools must be tailored to the end-user’s domain expertise. Furthermore, hybrid ML architectures demonstrate that clinical deployment requires not only high accuracy but also explicit validation of real-world effectiveness and safety [39], a principle equally applicable to pharmacovigilance ML systems.

Despite the immense potential of machine learning in mRNA vaccine pharmacovigilance, its clinical translation faces numerous challenges. Data quality and standardization are primary obstacles: multi-source real-world data exhibit high heterogeneity, numerous missing values, and inconsistent coding standards, necessitating the establishment of a unified data governance framework. Model interpretability is crucial for clinical acceptance: black-box models struggle to gain the trust of clinicians. The application of explainable artificial intelligence (XAI) methods, particularly SHAP (SHapley Additive exPlanations), which provides a unified game-theoretic framework for interpreting model predictions [40], and LIME (Local Interpretable Model-agnostic Explanations), which generates locally faithful explanations for any classifier [41], is increasingly important for translating ML predictions into clinically actionable insights. Regulatory approvals are also a bottleneck: currently, no machine learning-driven pharmacovigilance system has been officially approved by the FDA or EMA, and regulatory standards urgently need to be improved. Furthermore, issues of fairness and bias cannot be overlooked. Machine learning models may perform poorly in specific populations (such as minority groups or patients with rare diseases), leading to health inequities. Ensuring the representativeness of training data and the broadness of model validation are prerequisites for achieving fair pharmacovigilance.

## Limitations and future directions

This review has the following limitations. Firstly, this systematic review was not prospectively registered in PROSPERO or a similar registry. We acknowledge this as a methodological limitation. Secondly, the majority of the included studies were retrospective in design, lacking large-scale prospective validation, which may introduce selection bias and confounding factors. Thirdly, the small number of eligible quantitative studies and their methodological diversity precluded statistical pooling; findings are therefore descriptive rather than definitive. Fourthly, most studies did not undergo external validation, making generalizability uncertain; this is particularly concerning given the well-documented degradation of ML performance when models are transported across health systems. Fifthly, multi-source real-world data exhibit differences in collection standards, variable definitions, and population representation, necessitating careful interpretation. Additionally, the search strategy covered English-language databases only, potentially missing non-English publications or grey literature. Finally, post-marketing surveillance data for tumor mRNA vaccines and sa-mRNA vaccines remain limited, so ML applications in these fields are largely conceptual.

Future research should prioritize exploring multimodal data fusion and federated learning strategies. Federated learning techniques have already been validated in medical contexts for achieving cross-institutional collaborative modeling without sharing raw data [9], providing a methodological foundation for the integration of privacy protection for electronic health records, vaccine adverse reaction reporting systems, and wearable device data [42]. The feasibility of this approach has been demonstrated at scale: Dayan et al. successfully deployed federated learning across 20 hospitals from three continents to predict clinical outcomes in COVID-19 patients, proving that collaborative modeling can achieve performance comparable to centralized data aggregation while maintaining strict data governance [43]. By adapting globally trained risk models based on structured clinical data to local real-time physiological signal monitoring or using social media-validated early symptom signals to optimize risk stratification algorithms for electronic health records, it is possible to construct a “global-local” (glocal) drug surveillance network that combines global monitoring breadth with local response precision. Furthermore, the introduction of explainable artificial intelligence (XAI) methods has the potential to break the “black box” dilemma of machine learning models by quantifying the contributions of each feature to predictions using SHAP, LIME, and other techniques, thereby enhancing clinicians’ trust and acceptance of model decisions. For emerging platforms such as sa-mRNA vaccines and tumor mRNA vaccines, developing time series analysis and survival analysis methods is necessary to capture delayed adverse events, and exploring “small sample learning” and “transfer learning” strategies is essential to address the challenges posed by individualized vaccines. Transfer learning has been successfully applied in biomedical risk detection to leverage knowledge from source-domain signal data to improve prediction in target domains with limited labeled examples [44]. For sa-mRNA and tumor mRNA vaccines, models pre-trained on large COVID-19 vaccine safety datasets could be fine-tuned on smaller platform-specific cohorts, accelerating the development of clinically actionable risk predictors without requiring de novo training on prohibitively small datasets. Ultimately, establishing mechanisms for continuous model performance monitoring and adaptive updates will be crucial for ensuring the long-term reliability of algorithms in the face of dynamic changes such as viral mutations and vaccine formulation updates, thus enabling the transition of intelligent drug surveillance systems from concept to clinical application.

## Conclusion

Machine learning-assisted mRNA vaccine pharmacovigilance represents a profound shift from passive monitoring to active, intelligent monitoring. By integrating multi-source real-world data, machine learning technologies not only enhance the ability to detect rare adverse events early but also optimize clinical resource allocation through precise risk stratification. Despite challenges related to data quality, model interpretability, and regulatory approvals, as technology evolves and methodologies improve, an intelligent, precise pharmacovigilance system for vaccines will become a crucial infrastructure for safeguarding public health. In the future, interdisciplinary collaboration (clinical medicine, data science, regulatory science) will be key to driving progress in this field.

## Electronic Supplementary Material

Below is the link to the electronic supplementary material.

Supplementary material 1

## Data Availability

All data analysed in this systematic review are derived from publicly available published studies. The detailed search strategy, database sources, and study selection criteria are provided in the Methods section to ensure full reproducibility. No new primary datasets were generated during the current study.

## Abbreviations

BAPC: Bayesian Age-Period-Cohort
EAPC: Estimated Annual Percentage Change
CKD: Chronic Kidney Disease
LDL: Low-Density Lipoprotein
HDL: High-Density Lipoprotein
UI: Uncertainty Interval
AI: Artificial Intelligence
ML: Machine Learning
COVID-19: Coronavirus Disease 2019
EHR: Electronic Health Record
VAERS: Vaccine Adverse Event Reporting System
NLP: Natural Language Processing
FDA: Food and Drug Administration
PRR: Proportional Reporting Ratio
ROR: Reporting Odds Ratio
XAI: Explainable Artificial Intelligence
SHAP: SHapley Additive exPlanations
LIME: Local Interpretable Model-agnostic Explanations
EMA: European Medicines Agency
RSV: Respiratory Syncytial Virus
sa-mRNA: Self-amplifying Messenger Ribonucleic Acid
GMT: Geometric Mean Titer
irAEs: Immune-related Adverse Events
QUADAS-2: Quality Assessment of Diagnostic Accuracy Studies 2
AUC: Area Under the Curve
SMOTE: Synthetic Minority Over-sampling Technique
BERT: Bidirectional Encoder Representations from Transformers
BioBERT: Biomedical Bidirectional Encoder Representations from Transformers
CDC: Centers for Disease Control and Prevention
VSD: Vaccine Safety Datalink
RWD: Real-World Data
RCT: Randomized Controlled Trial
